# Molecular Characterization of a Lysozyme Gene and Its Altered Expression Profile in Crowded Beet Webworm (*Loxostege sticticalis*)

**DOI:** 10.1371/journal.pone.0161384

**Published:** 2016-08-30

**Authors:** Hailong Kong, Min Lv, Nian Mao, Cheng Wang, Yunxia Cheng, Lei Zhang, Xingfu Jiang, Lizhi Luo

**Affiliations:** 1 College of Horticulture and Plant Protection, Yangzhou University, Wenhui East Road, NO. 48, Yangzhou, 225009, China; 2 Institute of Agricultural Sciences of Lixiahe District in Jiangsu Province, Yangzijiang Road, NO. 568, Yangzhou, 225007, China; 3 State Key Laboratory for Biology of Plant Diseases and Insect Pests, Institute of Plant Protection, Chinese Academy of Agricultural Sciences, Yuanmingyuan West Road, NO. 2, Beijing, 100193, China; USDA Agricultural Research Service, UNITED STATES

## Abstract

There is growing evidence that insects living in high-density populations exhibit an increase in immune function to counter a higher risk of disease. This phenomenon, known as density-dependent prophylaxis, has been experimentally tested in a number of insect species. Although density-dependent prophylaxis is especially prevalent in insects exhibiting density-dependent phase polyphenism, the molecular mechanism remains unclear. Our previous study demonstrated that the antibacterial activity of lysozyme is important for this process in the beet webworm *Loxostege sticticalis*. In this study, a lysozyme cDNA from *L*. *sticticalis* was cloned and characterized. The full-length cDNA is 1078 bp long and contains an open reading frame of 426 bp that encodes 142 amino acids. The deduced protein possesses structural characteristics of a typical c-type lysozyme and clusters with c-type lysozymes from other Lepidoptera. *LsLysozyme* was found to be expressed throughout all developmental stages, showing the highest level in pupae. *LsLysozyme* was also highly expressed in the midgut and fat body. Elevated *LsLysozyme* expression was observed in *L*. *sticticalis* larvae infected by *Beauveria bassiana* and in larvae reared under crowding conditions. In addition, the expression level of *LsLysozyme* in infected larvae reared at a density of 10 larvae per jar was significantly higher compared to those reared at a density of l or 30 larvae per jar. These results suggest that larval crowding affects the gene expression profile of this lysozyme. This study provides additional insight into the expression of an immune-associated lysozyme gene and helps us to better understand the immune response of *L*. *sticticalis* under crowding conditions.

## Introduction

The immune system is very important to host defense against pathogens and parasites. Insects lack acquired immunity and thus are dependent innate immunity, which involves cellular and humoral immune responses [1]. The humoral immune response is based on antimicrobial peptides such as attacin, lysozyme, defensin and aecropin [2]. Lysozyme was the first antimicrobial peptide to be purified and was reported to play a critical role in the host immune response [3]. Lysozyme enzymes are a type of hydrolase that digests bacterial cell walls by cleaving the beta-1,4-glycosidic linkage between N-acetyl-D-glucosamine and N-acetylmuramic acid and hydrolyzes the β-1,4-linkage of chitooligosaccharides in fungal cell walls [4, 5]. There are six major lysozymes types reported to date, including chicken (c-type), goose (g-type), inverterbrate-type (i-type), plant, phage and bacterial [6].

Lysozyme plays a major role in insect defense against microorganisms, and several of these genes have been identified by global gene expression analysis after microbial challenge. For example, in the coleopteran *Tribolium castaneum*, mRNA levels for two lysozyme genes increased markedly following microbial challenge [7]. In the lepidopteran *Bombyx mori*, lysozyme genes are constitutively expressed but are up-regulated upon bacterial infection [8]. The antimicrobial activity of a recombinant lysozyme protein from the cricket *Gryllotalpa orientalis*, order Orthoptera, has been demonstrated by radial diffusion methods and minimal inhibitory concentrations [9]. In addition, immunological challenge resulted in increased levels of lysozyme activity in the fat body, hemocyte and hemolymph plasma of naïve and immunologically challenged desert locusts (*Schistocerca gregaria*) [10].

The beet webworm *Loxostege sticticalis* L. (Lepidoptera: Crambidae) is a primary insect pest of agriculture and livestock forage in northern China [11, 12], as well as in northern Asia, North America and Eastern Europe [13, 14], and outbreaks have seriously threatened agriculture and stockbreeding in the former [15, 16, 17, 18]. A characteristic of this species is periodic heavy outbreaks alternating with more or less long periods of high population density [19]. Insects living in high-density populations have evolved adaptive prophylaxis mechanisms to cope with pathogens and parasites [20], and due to facultative physiological resource allocation to defense mechanisms, species living under crowded conditions are predicted to be more resistant to natural enemies than those under low-density conditions. As this phenomenon, termed “density-dependent prophylaxis” [20], strongly affects host-parasite dynamics [21], understanding this response mechanism of a species is essential for characterizing its population dynamics and the potential for its control using natural enemies.

Our previous research suggested that *L*. *sticticalis* larvae exhibit density-dependent prophylaxis and that larval density could significantly enhance the antimicrobial activity of a larval lysozyme [22]. Previous research has demonstrated that the activity of lysozyme is significantly elevated in crowded populations [23]. Nonetheless, it remains unclear whether larval density can influence lysozyme at the transcriptional level and whether the lysozyme gene has a role in insect density-dependent prophylaxis. In this study, a full-length lysozyme cDNA from *L*. *sticticalis* was cloned and characterized, and mRNA expression was examined during the entire life cycle and in fifth-instar larvae reared under different population densities. The results advance our knowledge of immune-associated lysozyme gene expression and the density-dependent prophylactic response in *L*. *sticticalis*.

## Materials and Methods

### Ethics statement

*Loxostege sticticalis* individuals were initially collected in 2007 Siziwangqi, Inner Mongolia, China; a colony has been maintained in a room at the Institute of Plant Protection, Chinese Academy of Agricultural Sciences. No specific permit was required for the described field collection, and the location is not privately owned or protected in any way. Species of the family Crambidae are common agricultural pests and are not included in the ‘list of Protected Animals in China’.

### Experimental insect colony

A laboratory colony of *L*. *sticticalis* was initiated using diapausing larvae collected in 2007 in Siziwangqi, Inner Mongolia, China. The larvae were reared on *Chenopodium album* L. leaves at 22±1°C and 70% relative humidity, with an L: D photoperiod of 16: 8 h. When the larvae stopped feeding, sterilized soil containing 10% water was added to the cages to a depth of 10 cm to provide a substrate for cocoon formation, pupation, and adult emergence. Adult *L*. *sticticalis* individuals were placed in a 2-l-capacity plastic cage equipped with a cotton pad soaked in 10% glucose solution (w/v) and allowed to oviposit on the nylon gauze lining the page.

### Molecular cloning of lysozyme cDNA

According to the manufacturer’s instructions, total RNA was extracted from fifth-instar larvae of *L*. *sticticalis* using a Trizol Kit (Invitrogen, Carlsbad, CA, USA), and first-strand cDNA was synthesized using an oligo(dT)_15_ primer (Tiangen Biotech, China). Two primers (Lys-F1: 5’-TATCTGGAACAGGCCGTAGTC-3’; Lys-R1: 5’- TGCGCTAAGAAGATCTTCAAGCGCCAC-3’) were designed based on a highly conserved amino acid sequence (DYGL/IFQI) and (CAKKIF/YKRH) found in several insect c-type lysozymes. Using these primers and a 3’ anchor R (5’-GA), PCR amplification was carried out as follows: one cycle at 95°C for 3 min, 35 cycles at 95°C for 30 s, 60°C for 30 s, and 72°C 45 s, and an additional extension at 72°C for 10 min. The PCR products were purified using a gel purification kit according to the manufacturer’s instructions (Shenergy Biocolor, Shanghai, China), followed by ligation into the pMD-18T vector (Takara, Dalian, China) and transformation into competent DH5α cells. Positive recombinants were identified by blue-white color selection on ampicillin-containing LB plates, and PCR screening was carried out using the two primers described above. Positive clones were sequenced by Sangon Company (Shanghai, China).

To obtain the full-length cDNA sequence, 3’ and 5’ RACE reactions were performed using the primers including Lys-F1 and poly-T primer: 5’-GCGAATTCGTCGACAAGC(T)_17_−3’ and Lys-R1 and T-amp primer: 5’-GCGAATTCGTCGACAAGC-3’. Both PCR cycling parameters were 95°C for 3 min, 35 cycles at 95°C for 30 s, 60°C for 30 s, and 72°C 45 s, and an additional extension at 72°C for 20 min.

### Bioinformatic analysis

Sequence comparisons were carried out using the BLAST software (http://www.ncbi.nlm.nih.gov). The nucleotide sequence was translated into the amino acid sequence using the ExPaSy software (http://www.expasy.org). SignalP was employed for signal peptide prediction (http://www.cbs.dtu.dk/services/SignalP/). To investigate the phylogenetic relationship between *LsLysozyme* and lysozymes from other insect species, the amino acid sequences were aligned using the cluster algorithm ClustalW ver. 1.8, and a phylogenetic tree was constructed based on alignment by MEGA 4 [24].

### Development stage and tissue expression profiles

Total RNA was extracted from different development stages and different tissues of *L*. *sticticalis* using a Trizol Kit, as described above. The fat body, midgut and epithelial layer from five one-day-old fifth-instar larvae were dissected in DEPC-treated water. The hemolymph was collected from first abdominal proleg using a syringe. The hemolymph from twenty larvae was pooled together as one sample and immediately centrifuged at 800 × g and 4°C for 10 min for hemocyte collection. The epithelial layer, fat body and midgut were isolated, washed, and homogenized in an ice-bath using a Dounce homogenizer. After centrifugation (10, 000 ×, 10 min, 4°C), the supernatant was kept at -70°C for later use. The purified RNA was treated with RNase-free DNase (Promega, Madison, WI, USA) to remove the genomic DNA. The concentration of the purified RNA was measured using a Nanodrop spectrophotometer (Thermo Scientific, Waltham, MA, USA). First-strand cDNA was synthesized using an Oligo(dT)_15_ primer (Tiangen Biotech, China) and 1μg of total RNA as the template in a final volume of 10 μl. cDNA prepared from total RNA was used as the template for real-time quantitative PCR (qPCR) with an ABI PRISM 7500 Real-Time PCR System (Applied Biosystems Foster, CA, USA). *LsLysozyme* primers (F: 5’-TTTGGTTGGAGAGTGATGCT-3’; R: 5’-CGTGGTTCTACCGCTTTCAT-3’) were designed; 18 *S RNA* gene was selected as the reference gene (F: 5’- GCCGTTCTTAGTTGGTGGAG-3’; R:5’-AGTGAGTTGCGTGACAGAGC-3’).

Three biological replications were used for qPCR analysis using a 20 μl total reaction system containing 0.1 μg total RNA, 0.8 μl primer mix containing 10 μM of each forward and reverse gene-specific primers, 0.4 μl ROX Reference Dye-II (50×), 2 μl cDNA, 10 μl SYBR Premix EX TaqTM II and 6 μl H_2_O, following the One Step SYBR Premix Ex TaqTM II Kit instructions (Takara Biotechnology Dalian Co., Ltd). The thermal cycling conditions were 15 min at 95°C, followed by 40 cycles of denaturation at 95°C for 30 s, annealing at 58°C for 30 s and elongation at 72°C for 35 s. Relative quantifications were calculated using the 2^-ΔΔCt^ method [25].

### Expression profiles at different densities and after entomopathogenic fungus challenge

Expression of the lysozyme gene was evaluated in *L*. *sticticalis* larvae reared at different densities. After hatching, larvae were reared at three density treatments of 1, 10 and 30 larvae per 650-ml-capacity jar (diameter 10 cm). The number of larvae for the two higher density treatments was maintained at a constant level throughout the feeding period. Excess food was provided by adding fresh leaves every morning. All insects were maintained under the same condition as described above the colony population. The hemolymph was collected as described above when most of the larvae at a given density had developed to the second day of the fifth instar, and the samples collected from groups of 20 larvae per density were pooled as one replicate. Three replicates were employed for each density. RNA isolation, first-strand cDNA synthesis, and qPCR were performed as described above.

The expression profiles of the lysozyme gene were also evaluated in *L*. *sticticalis* larvae reared at different densities and exposed to the entomopathogenic fungus *Beauveria bassiana* obtained from Shenwei Biological Company (Yancheng, Jiangsu, China). Fungal spores were suspended in sterile 0.1% Tween 80 solution (polysorbate), and the final concentration was adjusted to 1.6 × 10^7^ conidia ml^-1^ for infection. When most of the larvae at a given density had developed to the fifth instar, a group of ten larvae per treatment was exposed to the fungus by dipping them together in the spore solution for 5 s followed by air-drying at room temperature. A corresponding control group was dipped in the same way in sterile 0.1% Tween 80 solution only. After dipping, all exposed and control larvae were individually maintained in small glass jars under the same environmental conditions as described above. After 24 h, the larvae were used for hemolymph collection. Three replicates were employed for each density. The hemolymph treatment and qPCR procedure were the same as described above.

### Statistical analysis

Data for developmental stage and tissue and density-dependent lysozyme gene expression were tested using one-way analysis of variance (ANOVA). Data for lysozyme gene expression before and after treatment with *B*. *bassiana* were subjected to Student’s *t* test. All statistical analyses were performed with SPSS 10.0.

## Results

### Molecular cloning of *LsLysozyme*

**Fig 1:**
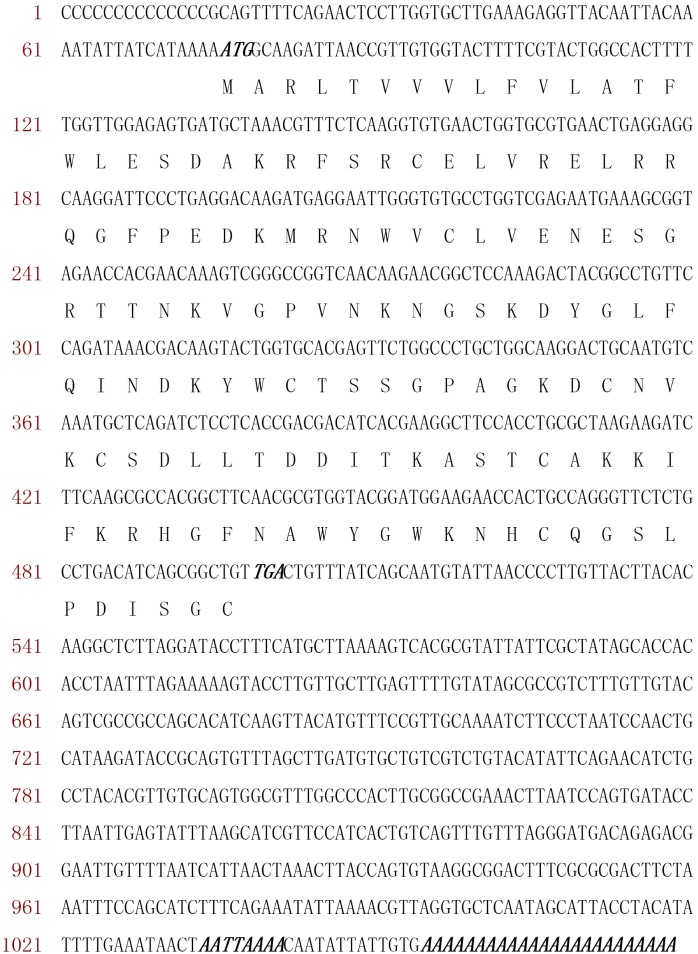
Nucleotide and deduced amino acid sequences of *L*. *sticticalis* lysozyme. The nucleotides are numbered on the left from the first base of the cDNA. The deduced amino acid sequence is shown below the nucleotide sequence in single-letter code. The codons for initiation, termination, polyadenylation and the poly (A) tail are indicated in bold-italic.

The c-type *LsLysozyme* cDNA amplified from *L*. *sticticalis* is 1096 bp long and contains an open reading frame (ORF) of 426 bp that encodes a protein of 141 amino acids. The ATG start codon is found at base pairs 76–78 and the in-frame TGA stop codon at 499–501, followed by the polyadenylation signal sequence “AATTAAAA” and poly(A) at the 3’-untranslated region (Fig 1). The ORF was composed of 21 residues at the N terminal side predicted as a signal peptide and 120 residues as a mature peptide. The molecular mass of mature *LsLysozyme* protein (amino acids 22 through 141) is predicted to be 13.6 kDa, with a calculated isoelectric point of 9.19. A search of the Pfam database revealed that the *L*. *sticticalis* lysozyme homolog contains a typical alpha-lactalbumin/lysozyme C domain from position 22 to 141 (http://pfam.sanger.ac.uk/).

### Comparison of the *LsLysozyme* amino acid sequence with those of lysozymes from other insects

**Fig 2:**
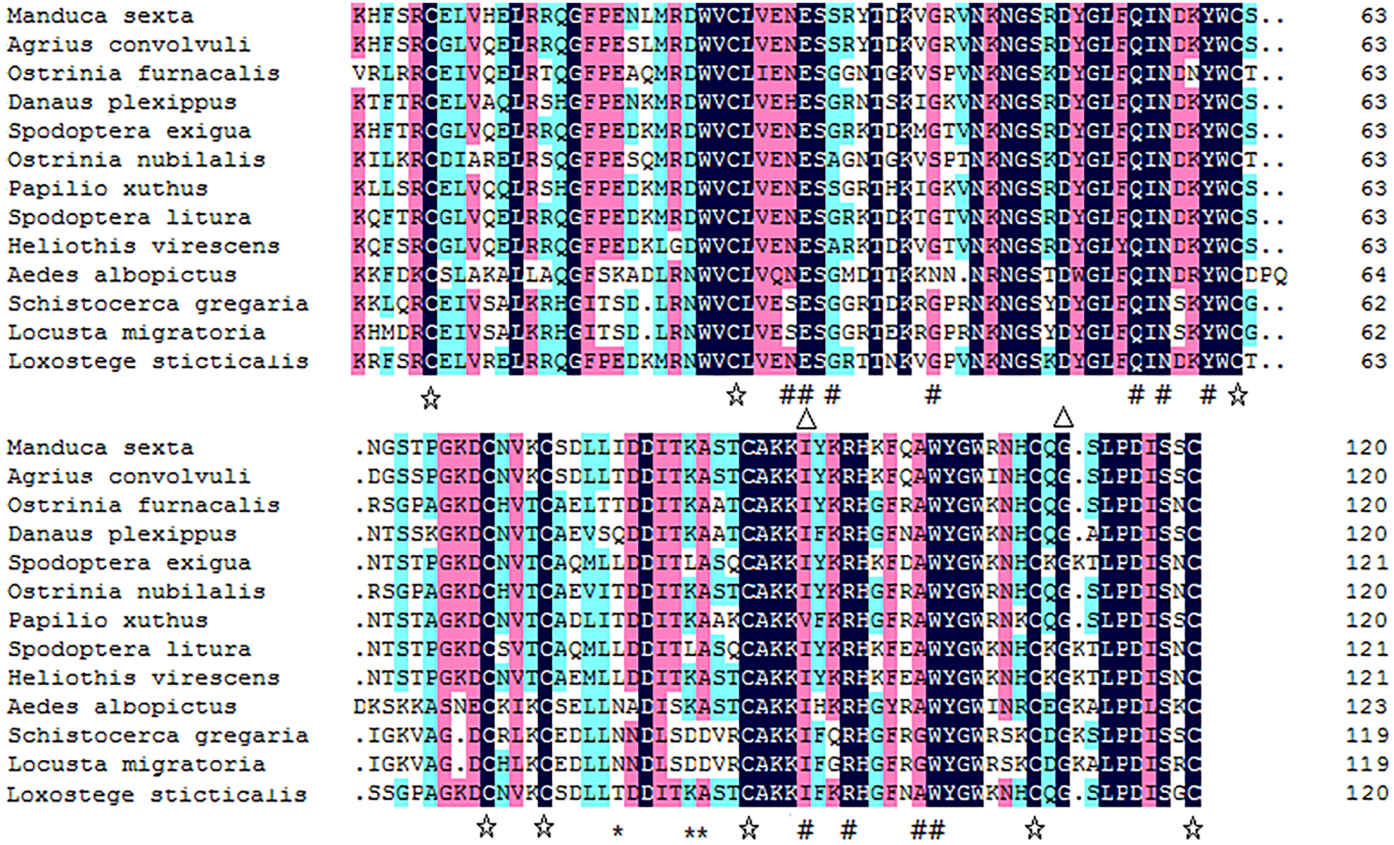
Multiple sequence alignment of beet webworm lysozyme C with other Lepidoptera insect lysozymes. The star symbols (☆) mark conserved cysteine residues; The 11 residues that compose the lysozyme catalytic cleft on the conserved LYZ1 domain are indicated with triangle (△). The two major residues, Glu^53^ and Asp^71^, responsible for the puptative lysozyme catalytic site on conserved LYZ1 domain are marked with numbersign (#). Asterisks (*) indicate Ca^2+^ binding site on conserved domain LYZ1.

Using a BLAST search, *LsLysozyme* was shown to have high identity with other lepidopterans (Fig 2). For example, the protein exhibits the following similarity: 77% with *Manduca sexta* lysozyme; 76% with *Agrius convolvuli* lysozyme, *Ostrinia furnacalis* lysozyme 6 and *Ostrinia nubilalis* lysozyme; 74% with *Pieris rapae* lysozyme II; 70% with *Pseudoplusia includens* lysozyme; and 71% with *Spodoptera exigua* lysozyme.

Multiple sequence alignment of mature *LsLysozyme* amino acid sequence of beet webworm with twelve mature lysozymes from other Lepidoptera insects was presented in Fig 2. According to the results, *LsLysozyme* has 8 conserved cysteine residues (Fig 2 ☆) (Cys^6^, Cys^26^, Cys^62^, Cys^72^, Cys^76^, Cys^90^, Cys^110^, Cys^120^). And it posses all features of a c-type lysozyme, for example, the two key residues, Glu^53^ and Asp^71^ of the catalytic site (Fig 2 #), other 11 residues Asn^52^, Glu^53^, Gly^55^, Gly^62^, Gln^76^, Asn^78^, Tyr^81^, Ile^116^, Arg^118^, Ala^123^, and Trp^124^ which formed the lysozyme catalytic cleft (Fig 2, △), and the resuides Thr^102^, Lys^107^, and Ala^108^ which were Ca^2+^ binding site (Fig 2, *).

**Fig 3:**
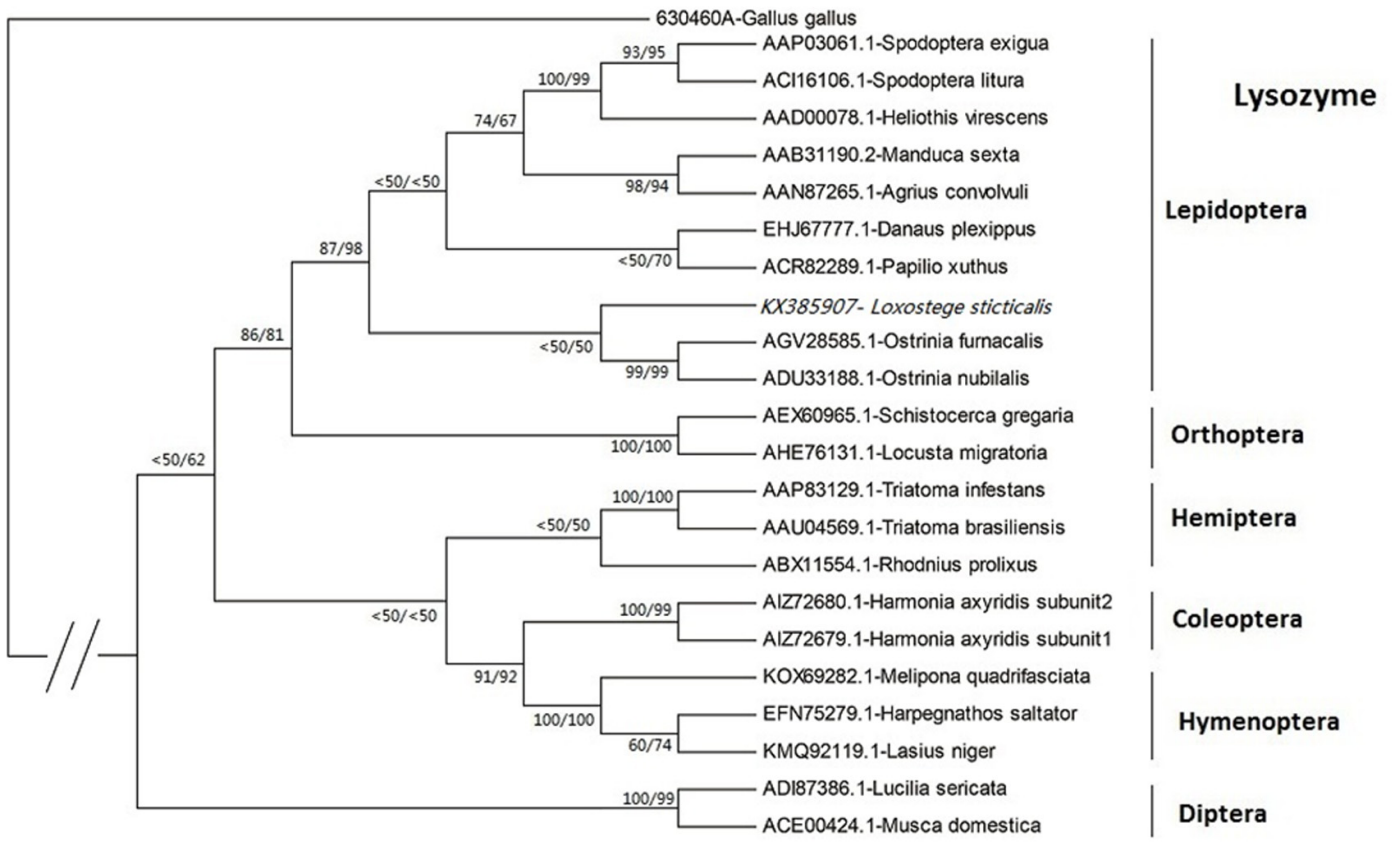
Phylogenetic tree of amino acid sequences of c-type lysozymes from different Insecta orders (Lepidoptera, Hemiptera, Hymenoptera, Coleoptera, Orthoptera and Diptera) was constructed by MEGA 4.0. *Gallus gallus* lysozyme was used as the out-group. Bootstrap values are indicated in maximum evolution and minimum likelihood for each root, respectively. *L*. *sticticalis* lysozyme is written italic letters.

A phylogeny tree was constructed in MEGA 4.0 using the amino acid sequences of c-type lysozymes from different Insecta orders and *Gallus gallus* was used as out-group (Fig 3). As shown, the insect c-type lysozymes are distributed in three main clades corresponding to Lepidoptera and Orthoptera lysozymes on one branch, and other insects (Hemiptera, Coleoptera and Hymenoptera) on the other branch. In addition, a group of lysozymes of cyclorrhaphan Diptera is also present. The *LsLysozyme* belong to the lysozyme of in the Lepidoptera and Orthoptera branch.

### Development and tissue expression analysis

**Fig 4:**
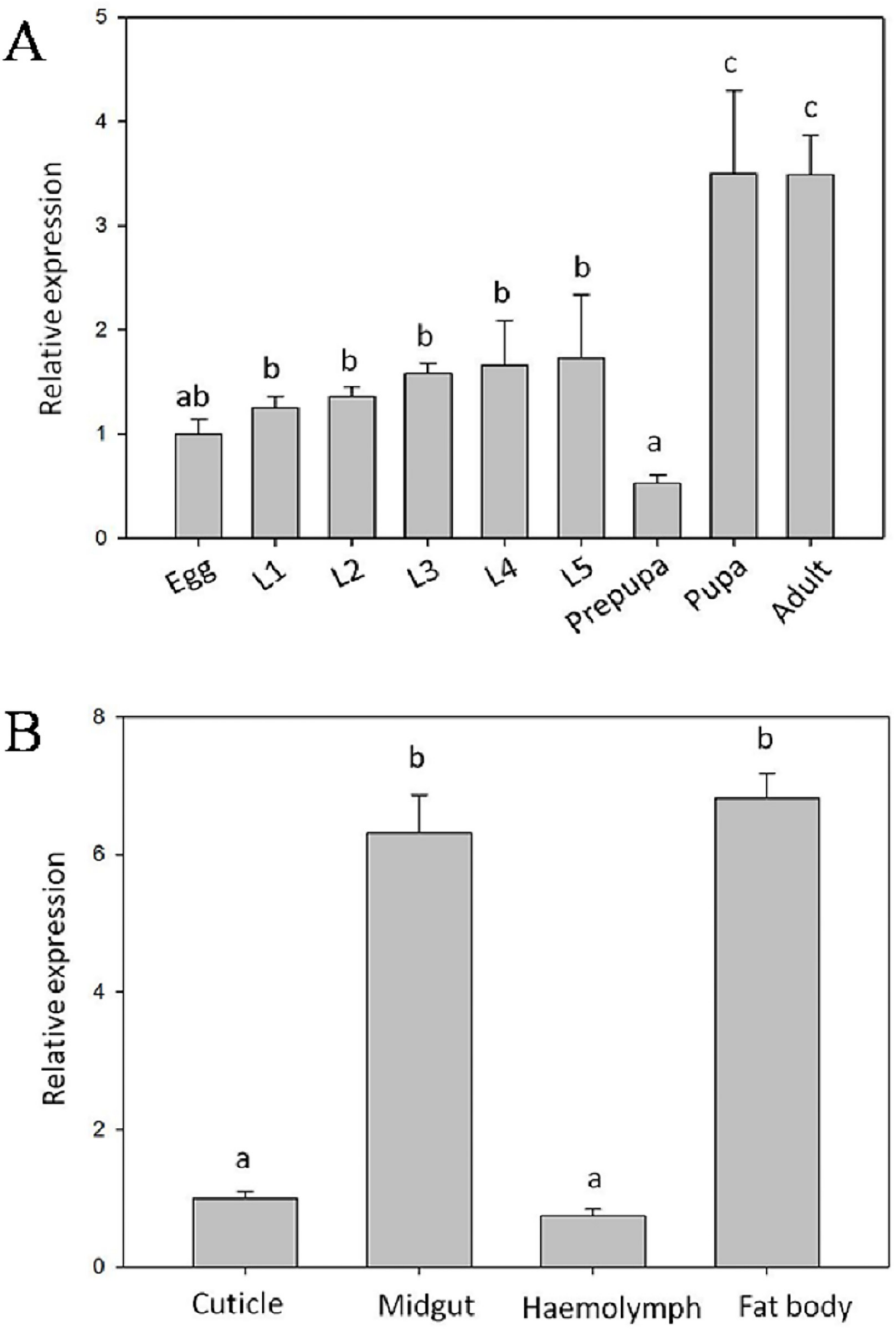
Expression patterns of *LsLysozyme* during life stages and in different tissues. The expression profiles of *LsLysozyme* during developmental stages are depicted in (A). L1-L5, 1st to 5th-instar larvae. The tissue expression distribution of *LsLysozyme* is shown in (B). Data are shown as the mean ± SE, n = 3. Different letters indicate significant difference at P < 0.01.

The results of qPCR assay showed that *LsLysozyme* was expressed during all *L*. *sticticalis* developmental stages, with lower expression during the embryonic stage. *LsLysozyme* became up-regulated (P < 0.01) at the first instar, and this high level was maintained through the fifth instar before declining in the prepupal stage and then increasing again in the pupal stage (Fig 4A).

The expression level of *LsLysozyme* was highest in the fat body (P < 0.01), at approximately 6.82-, 1.08-, and 9.22-fold higher than that in the cuticle, midgut and hemolymph, respectively (Fig 4B).

### Expression analysis of *LsLysozyme* from larvae reared under different densities before and after infection with the entomopathogenic fungus *B*. *bassiana*

**Fig 5:**
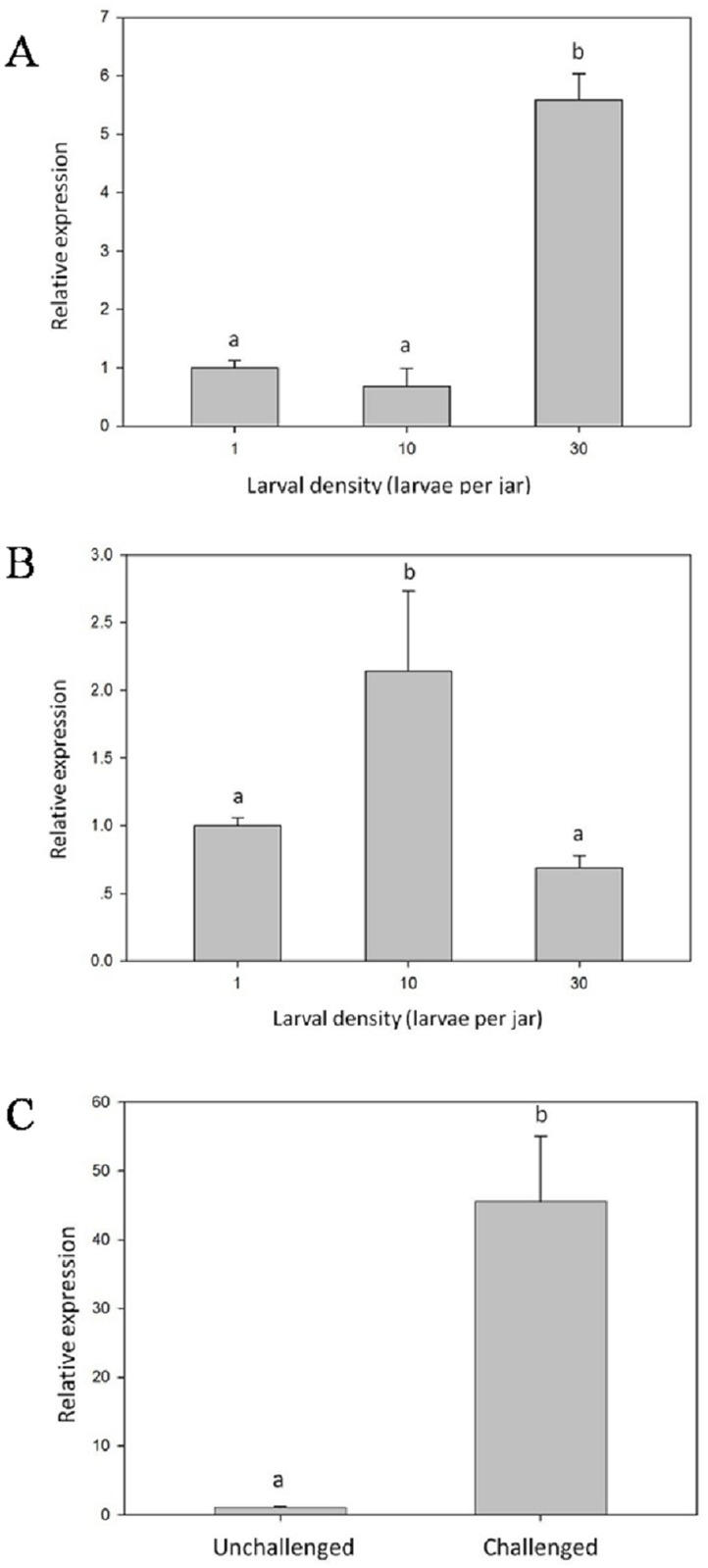
The relative gene expression of *LsLysozyme* among larvae reared at densities of 1, 10 and 30 larvae per jar and before and after treatment with *B*. *bassiana* are shown in A and B, respectively. The relative gene expression of *LsLysozyme* in fifth-instar larvae infected with *B*. *bassiana* is shown in C. Data are presented as the mean±SE, n = 3. Different characters denote significant difference at P < 0.01.

The expression level of *LsLysozyme* differed significantly among the three densities and was highest in larvae reared at a density of 30 larvae per jar (Fig 5A). Conversely, there was no significant difference in lysozyme expression between individually reared larvae and those reared at a density of 10 larvae per jar. When the larvae were infected by *B*. *bassiana*, the expression pattern of *LsLysozyme* among the three densities changed (Fig 5B). Infected larvae reared at the density of 10 larvae per jar displayed the highest expression level. The expression level of infected larvae reared at the density of 10 larvae per jar was significantly higher than that of larvae reared at a density of l or 30 larvae per jar, though there was no significant difference between the latter two treatments. The expression level of *LsLysozyme* in larvae increased significantly at 24 h after *B*. *bassiana* infection (Fig 5C).

## Discussion

A c-type lysozyme cDNA was amplified from an important agricultural pest, the beet webworm *L*. *sticticalis*. The mature *LsLysozyme* contains an ORF of 426 bp that encodes a 121-amino acid protein (from amino acids 22 to 141). The alignment multiple sequences between the *L*. *sticticalis* and other Lepidoptera mature c-type lysozyme amino acid shows that *LsLysozyme* is highly conserved in these Lepidoptera c-type lysozymes including the 8 cysteine residues. The high conservation cysteine residues indicate their importance in the formation of disulfide bridges and three dimensional structure of the molecule. There are three features in a typical c-type lysozyme. For example, Glu^53^ and Asp^71^ of the catalytic site, 11 residues Asn^52^, Glu^53^, Gly^55^, Gly^62^, Gln^76^, Asn^78^, Tyr^81^, Ile^116^, Arg^118^, Ala^123^, and Trp^124^ which formed the catalytic cleft, and the resuides Thr^102^, Lys^107^, and Ala^108^ which were the Ca^2+^ binding site (Fig 2). Especially, Glu^53^ and Asp^71^ of the catalytic site were fundamental for the biological activity of the lysozymes [26]. One major difference in the active site residues in terms of hydrophobicity between *LsLysozyme* and the other two Orthoptera insects lysozymes occurs at Ala^100^ (hydropathy index: 1.8) of *LsLysozyme*, which is Gly (hydropathy index: -0.4) in two Orthoptera insects lysozymes.

The active site of HEWL included 6 subsites from A to F which are able to bind 6 sugar residues [27, 28]. These subsites are present in the *LsLysozyme* as Asn^31^, Gly^34^, Tyr^60^, Trp^61^, Arg^97^, and Trp^103^(Fig 2) and are forecasted to interact with sugar rings placed in the subsites E, F, B, C, A and D, respectively. The substitution of the conserved residues with others was supposed to modulate the lysozyme activity. For example, when Asn^37^ and Trp^62^ of HEWL were replaced by Gly^33^ and Tyr^59^ of *S*. *gregaria*, respectively, exhibited about 2–3 fold higher bacteriolytic activity than HEWL [29]. As a matter of fact, such substitution also occurred naturally in *LsLysozyme*, since the sequence of *LsLysozyme* also contains a Gly in positon 34 and a Tyr in position 60, an enhanced bacteriolytic activity may be observed in *L*. *sticticalis* than HEWL.

One 7-peptide motif (GI/LF/YQIND/N) and two 3-peptide motifs (N/DGS, Y/FWC) are conserved among C-type lysozymes [5], and they are also found in the lysozyme from *L*. *sticticalis*. Together, these sequence motifs as well as the alignment and phylogenetic results confirm that *L*. *sticticalis* lysozyme belongs to the C-type lysozyme family of proteins. C-type lysozymes are divided into two major types, non-calcium binding and calcium binding, and *L*. *sticticalis* lysozyme C appears to belong the latter.

Lysozyme was found to be constitutively expressed during the entire life cycle of *L*. *sticticalis*, and the expression level increased constantly during the larval stage, which was accord with the expression pattern in *Anopheles dirus* [27] [30]. A similar expression profile of lysozyme was also reported for the housefly *Musca domestica* [5]. Lysozyme expression in *L*. *sticticalis* was detected at lower levels in prepupae, medium levels in larvae and higher levels in adults, peaking in pupae. The highest expression level during the pupal stage was also found in *Helicoverpa armigera* [28] [31]. Such significant changes in lysozyme expression may be partially explained by the complete reorganization of body tissues during the transition from larva to adult. However, the needs to be elucidated by further study.

*L*. *sticticalis* lysozyme is expressed in all tissues, with high mRNA levels in the fat bodies and midgut and low mRNA levels in the epidermis and hemolymph. This expression pattern is similar to *Ostrinia nubilalis* lysozymes, which are also expressed in the epidermis, fat body, midgut and hemolymph [32]. Except for its role in antimicrobial defense, lysozyme has been reported to have a digestion role in some Diptera and Hemiptera [32, 33]. The phylogenetic analysis based on the deduced amino acid sequences showed that lysozyme from *L*. *sticticalis* is grouped with those molecules from Lepidoptera, and far from that of the cyclorrhaphan Diptera. Both maximum evolution and minimum likelihood analysis supported the above results. Lysozymes from Lepidoptera are immune-related, however, those from cyclorrhaphan Diptera are of digestive function [29, 34]. Therefore, *LsLysozyme* gene might play an important role in immunity rather than in digestive functions.

As expression of the *L*. *sticticalis* lysozyme gene was up-regulated when larvae were challenged with *B*. *bassiana* (Fig 5C), this enzyme may play an important role in *L*. *sticticalis* larval defense against this fungus. Prior to infection with *B*. *bassiana*, the expression level of the gene in larvae reared at the density of 30 larvae per jar was significantly higher than that in larvae reared at densities of 1 and 10 larvae per jar. The results of our previous study on the antimicrobial activity of lysozyme in *L*. *sticticalis* larvae also showed significantly higher activity in larvae at a density of 30 larvae per jar compared to larvae reared individually [22]. This expression pattern may be caused by larval crowding. Prophylaxis in *Anticarsia gemmatalis* larvae is reported to be triggered by the presence of conspecifics [35], and when the larval density reached 30 larvae per jar, the crowded conditions up-regulated lysozyme expression, increasing prophylaxis.

After larvae were infected by *B*. *bassiana*, the expression level of *LsLysozyme* of larvae reared at a density of 10 larvae per jar was significantly higher than that of larvae reared at densities of 1 and 30 larvae per jar. However, this pattern is different from the expression pattern of non-immunized larvae before infection. The difference may be related to variation in *LsLysozyme* gene expression in infected versus non-infected larvae, as expression was significantly increased when the larvae were infected with *B*. *bassiana* (Fig 5C). Transcripts of lysozyme c-1 and c-2 genes were also significantly increased after immune challenge with *Escherichia coli* or *Micrococcus luteus* [36]. Therefore, infection with *B*. *bassiana* may have influenced the expression pattern of the lysozyme gene in larvae reared at the three different densities.

We identified significant transcriptional up-regulation of lysozyme expression in the larval hemolymph in response to larval rearing density. And increased phenoloxidase activity, total haemocyte count and lysozyme activity were found in the crowded *L*. *sticticalis* [22]. So, defense strategy for enhancing specific effectors (such as lysozyme) by activate the entire immune pathways might be taken in the Lepidoptera *L*. *sticticalis*. However, this defense strategy may be different from the Orthoptera *Locusta migratoria*. Wang et al. (2013) [37] found that gregarious (crowded) migratory locusts exhibited high level of circulation PRPs but not AMPs, and there were no significantly differences in the PO activity, encapsulation response, and total hemocyte count between gregaria and solitaria locusts [23]. These results suggested a selection of other tolerance strategy for inhibiting pathogen spread and for increasing the “distance” between infected and susceptible individuals that together improved the immune defense of gregarious locusts [37].

This is the first report of the isolation and characterization of a c-type lysozyme from *L*. *sticticalis*. These results suggest that larval crowding could increase lysozyme gene expression. Therefore, larvae may use crowding as a cue to induce lysozyme expression to protect against possible infection. To better understand the immune function of *LsLysozyme* among larvae reared at different densities, the activity and expression of the protein in response to infection should be further analyzed. Additional research involving functional characterization of population density-regulated genes would reveal the precise gene pathways and regulatory mechanism of density stress at the molecular level.
